# Centromeric Non-Coding RNAs: Conservation and Diversity in Function

**DOI:** 10.3390/ncrna6010004

**Published:** 2020-01-17

**Authors:** Takashi Ideue, Tokio Tani

**Affiliations:** Department of Biological Sciences, Faculty of Advanced Science and Technology, Kumamoto University, Kumamoto 860-8555, Japan; ttani@kumamoto-u.ac.jp

**Keywords:** centromere, chromosome segregation, RNP

## Abstract

Chromosome segregation is strictly regulated for the proper distribution of genetic material to daughter cells. During this process, mitotic chromosomes are pulled to both poles by bundles of microtubules attached to kinetochores that are assembled on the chromosomes. Centromeres are specific regions where kinetochores assemble. Although these regions were previously considered to be silent, some experimental studies have demonstrated that transcription occurs in these regions to generate non-coding RNAs (ncRNAs). These centromeric ncRNAs (cenRNAs) are involved in centromere functions. Here, we describe the currently available information on the functions of cenRNAs in several species.

The centromere is a chromosomal region that is required for chromosome segregation in mitosis [1]. The transcripts of this region, known as centromeric RNAs (cenRNAs), are thought to play a role in centromere function in several species [2,3,4,5]. Energetic studies on numerous types of protein factors that are involved in chromosome and kinetochore functions have been conducted. In addition, RNAs derived from centromeres have been shown to be involved in centromere function. Therefore, determining how this type of RNA functions is necessary to completely understand regulation in this region.

Centromere protein A (CENP A) is a centromere-specific histone H3 variant. CENP A, containing a nucleosome, is thought to be the landmark for the centromeric region [1,6,7]. Therefore, elucidating the location of and the mechanism through which CENP A is incorporated into the nucleosomes is very important; however, these aspects are still not fully understood. Chromosome segregation requires the kinetochore to link the centromeric DNA to spindle microtubules [8]. More than 100 kinetochore proteins have been identified in humans [7]. CENP C is one of the fundamental kinetochore proteins [8]. It can bind directly to centromeric nucleosomes, DNA, and RNA. The association of CENP C with the nucleosomes is also important for defining centromere identity. The chromosome passenger complex (CPC), which consists of Aurora kinase B, INCENP, borealin, and survivin, regulates several mitotic processes [9]. CPC localizes at the centromere during pro-metaphase and metaphase periods. The recruitment of CPC to centromeres requires specific histone modifications in this region. Survivin binds to histone H3, which is phosphorylated at Thr 3 (H3T3). In contrast, borealin interacts with shugoshin (Sgo1) bound to phosphorylated histone H2A at Thr 120 (H2AT120) [10].

Prior investigations have revealed the roles of cenRNAs in CENP A loading [2,4,5,7,8,9], association of CENP C to kinetochore [11], and CPC localization to centromeres in several species [3]. These properties are very important for identifying the centromeres. In addition, cenRNAs are reported to be involved in the regulation of chromosome segregation. Although the centromere plays important roles in chromosome segregation, there are no sequence similarities in this region among different species. Therefore, cenRNAs derived from this region also have no common sequence and structure. Nevertheless, they all function at the centromere. We herein summarize the functions of cenRNAs and describe the similarities and differences among species.

## 1. Roles of Human cenRNAs in Centromere Function

Human centromeres consist of repetitive regions named satellite sequences. Their sizes range between 1 and 10 Mbp. The alpha-satellite (α-satellite) is a satellite repeat, in which one unit is 171 bp in length [6]. The centromere has been suggested to form heterochromatin and be inactive for transcription. However, the elongation of transcripts by RNA polymerase II (Pol II) has been detected at the centromeres in mitotic cells [12]. An α-amanitin treatment was shown to induce a reduction of RNAs at the centromeres and an increase at the lagging chromosomes. Under these conditions, the amounts of CENP C at the centromeric regions decreased. These findings suggest that transcription of the centromeric region is required for accurate mitotic progression [12].

Transcripts from the α-satellite, named satellite I non-coding RNAs (ncRNAs), are now drawing attention, as cenRNA has been shown to be involved in the regulation of chromosome segregation in HeLa cells [13]. Satellite I ncRNA does not contain poly-A tails and localizes at the centromeres. The mechanisms that retain satellite I ncRNA at the centromere have not yet been elucidated in detail [13]. The satellite I region is transcribed on both strands; thus, both sense and antisense cenRNA are detected [13]. However, it is unclear whether they have different roles as part of double-strand RNA.

**Figure 1:**
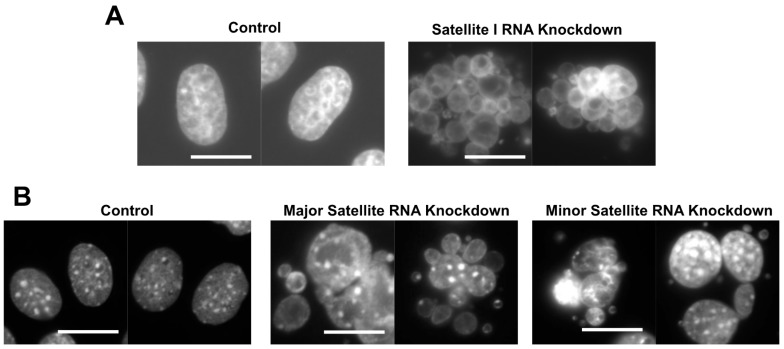
Knockdown of centromeric RNA (cenRNA) induces an abnormal nuclear morphology due to defects in chromosome segregation in human and mouse cells [13]. (**A**) Knockdown of satellite I RNA generates cells with abnormal nuclei exhibiting a grape-shape phenotype. Cells were stained with DAPI. Scale bar: 20 µm [13]. (**B**) Knockdown of major and minor satellite RNAs induces the grape-shape phenotype in mouse C2C12 cells. Scale bar: 20 µm [13].

The cenRNA knockdown in HeLa cells using antisense oligonucleotides induced the formation of an abnormal nuclear morphology, containing multiple small nuclei in a cell (Figure 1A) [13]. This phenotype is called the grape-shape phenotype. We observed the generation of this phenotype using HeLa cells expressing the histone 2B-GFP fusion protein by time-lapse. In normal mitosis, chromosomes are condensed, aligned, segregated, and then de-condensed within ~90 min. Contrastingly, in the antisense satellite I ncRNA oligo-introduced cells with the grape-shape phenotype, chromosomes were condensed, but did not align or segregate. After the misalignment of chromosomes at the cell equator plane, they finally de-condensed without segregation, resulting in the creation of multiple small nuclei. Therefore, this phenotype is induced by defects in chromosome segregation.

Immunoprecipitation (IP) analysis revealed that Aurora kinase B associates with satellite I ncRNA [13]. This protein is the key kinase of cell division and a component of CPC. In the satellite I ncRNA knockdown cells, the localization of Aurora kinase B was defective at the mitotic chromosomes, and its kinase activity was abnormally elevated. These findings suggest that satellite I ncRNA regulates chromosome segregation through control of the Aurora B function [13].

Several proteins have been identified as satellite I ncRNA-binding factors using pulldown and IP experiments [14]. Since some binding factors differ between interphase and mitotic phase complexes, the composition of the satellite I ribonucleoprotein (RNP) complex appears to change during cell cycle progression [14].

RBMX/hnRNP G is a candidate binding protein for satellite I ncRNA [14]. Although the association of RBMX to satellite I ncRNA was confirmed by IP experiments, it was specific to the mitotic phase. The depletion of RBMX in HeLa cells causes defective chromosome segregation, similar to the satellite I ncRNA knockdown cells. RBMX has been reported to be a cohesion regulator [15]. The abnormal separation of sister chromatids has been observed in both RBMX and satellite I ncRNA-depleted cells. Therefore, satellite I RNP containing RBMX may function in chromosome segregation through the regulation of sister chromatid cohesion [14].

DHX38 is a splicing-related DEAH box helicase [16], which binds to satellite I ncRNA [17]. The depletion of DHX38 causes defects in chromosome segregation, similar to satellite I ncRNA knockdown [17]. Satellite I ncRNA accumulates throughout the cell cycle, and IP experiments have shown that DHX38 interacts with this RNA in the interphase. In contrast, this association was not observed in the mitotic phase. Interestingly, the depletion of DHX38 impaired the localization of Aurora kinase B to the mitotic chromosomes, suggesting that an impaired satellite ncRNP composition in the interphase affects the mitotic function of the ncRNP complex [17]. The fission yeast Prp16p, a homologue of human DHX38, interacts with yeast pericentromeric *dg* ncRNA to form heterochromatin in this region [18], indicating that the interaction between this protein and cenRNA is conserved in humans and fission yeast. DHX38 has also been reported to be a component of the interphase centromere (ICEN) complex [19]. This complex was identified by the IP of CENP A from nuclear extracts of HeLa cells in the interphase. Some CENPs were also found in this complex. The centromeric functions of the ICEN complex and DHX38 in the interphase currently remain unclear.

Transcripts from α-satellite repeats are also considered to be involved in CENP A recruitment. Active Pol II co-localizes with CENP A and B in the early G1 phase of the cell cycle [20]. The α-satellite ncRNA, 1300 nucleotides in length, was detected in immunoprecipitated samples of CENP A and its chaperone holiday junction recognition protein (HJURP), suggesting that they form a complex [20]. The downregulation of α-satellite ncRNA using small interfering RNAs (siRNAs) caused mitotic defects due to the reduction of CENP A and HJURP at the centromere [20].

In α-satellite repeats, there are active and inactive arrays for centromeric functions. Both arrays produce transcripts of 500~2000 nt. The number of transcripts from an active array is higher than that of an inactive one. RNA-DNA fluorescence in situ hybridization (FISH) showed that these transcripts are associated with the centromere in cis. Chromatin immunoprecipitation (ChIP) analysis revealed that CENP A co-precipitates with α-satellite ncRNAs derived from active arrays [21]. The target degradation of these RNAs resulted in cell cycle arrest before mitosis and reduced CENP A at centromeres [21], suggesting that cenRNA is essential for CENP A loading on the centromere.

The localization of Sgo1 is also affected by the centromeric transcription and transcripts. This protein prevents cohesion degradation at the centromeres until the segregation of chromosomes [22]. Therefore, the localization of Sgo1 to the inner centromere is critical for accurate segregation. The mechanism underlying the localization of Sgo1 to a proper position requires Pol II transcription at the centromere. Sgo1 binds to α-satellite RNA and Pol II. The inhibition of Pol II results in the redistribution of Sgo1 from the inner centromere to the kinetochore [23].

It has also been reported that transcripts from α-satellite repeats are processed into small RNAs [24]. However, whether a part of cenRNAs act as siRNAs remains unclear. In a chicken–human hybrid DT40 cell line that contained human chromosome 21, conditional loss-of-function of Dicer resulted in abnormal mitotic cells and showed premature sister chromatid separation [24]. This phenotype has been attributed to the aberrant accumulation of transcripts from α-satellite repeats of the human chromosome and abnormalities in the localization of heterochromatin proteins at the centromere. These observations showed the possibility of cenRNA being processed into small RNAs by Dicer to be involved in heterochromatin formation of the centromere. It should be determined whether human cenRNA functions as long or processed small RNAs.

The effects of the overexpression of α-satellite RNA remain controversial. The ectopic expression of seven repeats of satellite I units did not affect the nuclear morphology of hela cells [13]. Contrarily, cells transfected with lentiviral vectors expressing α-satellite RNA showed chromosomal instability due to segregation errors [25]. In the former case, cenRNAs were produced from plasmids, but in the latter, they were integrated into the chromosome. Overall, the effect of the ectopic expression of cenRNA in human cells continues to be controversial.

## 2. cenRNAs in Mice

cenRNA is also reported to be involved in the centromeric function in mice. The pericentromeric and centromeric regions of mice consist of two kinds of repetitive regions called major and minor satellites that contain 233-bp and 123-bp repeated units, respectively [26,27,28]. The sequences of these repeats have no similarity with humans. The length of mouse cenRNAs is also still unknown, along with the relevant promoter. Northern blot analysis using an anti-γ satellite (major satellite) probe revealed that the transcription of these regions depends on cell proliferation and the cell cycle [29]. A more abundant population of large and heterogeneous transcripts was detected in the late G1 phase and decreased during the mid-S phase. These transcripts were not detected in quiescent cells. In addition, a small RNA species was synthesized during the mitotic phase. Contrastingly, another group reported that the amount of minor satellite ncRNA peaks in the G2/M phase [30]. Therefore, the accumulation of mouse cenRNA throughout the cell cycle requires additional investigation.

The knockdown of major or minor satellite RNA in mouse C2C12 cells induced the grape-shape phenotype due to defects in chromosome segregation, similar to the satellite I ncRNA knockdown in HeLa cells (Figure 1B) [13]. RNA pulldown experiments revealed that mouse Aurora B, Survivin, and INCENP bind to minor satellite ncRNA [30]. RNase A treatment reduced the activity of Aurora B kinase. In vitro-transcribed minor satellite ncRNA rescued reduction of the kinase activity of Aurora B [30]. The enforced expression of minor satellite RNAs using an expression vector caused the mislocalization of Aurora B to the centromere and lack of sister chromatid cohesion [27]. These observations showed that Aurora B and the CPC complex are regulated by cenRNA in mice, similar to humans.

A pulldown experiment of minor satellite ncRNA revealed that mouse RBMX binds to minor satellite ncRNA [31]. RBMX has also been identified as an interaction factor of cenRNA in humans. This factor is involved in the regulation of sister chromatid cohesion on human cenRNA [14]. Therefore, it is important to determine whether RBMX has a similar function in mouse cenRNA.

**Figure 2:**
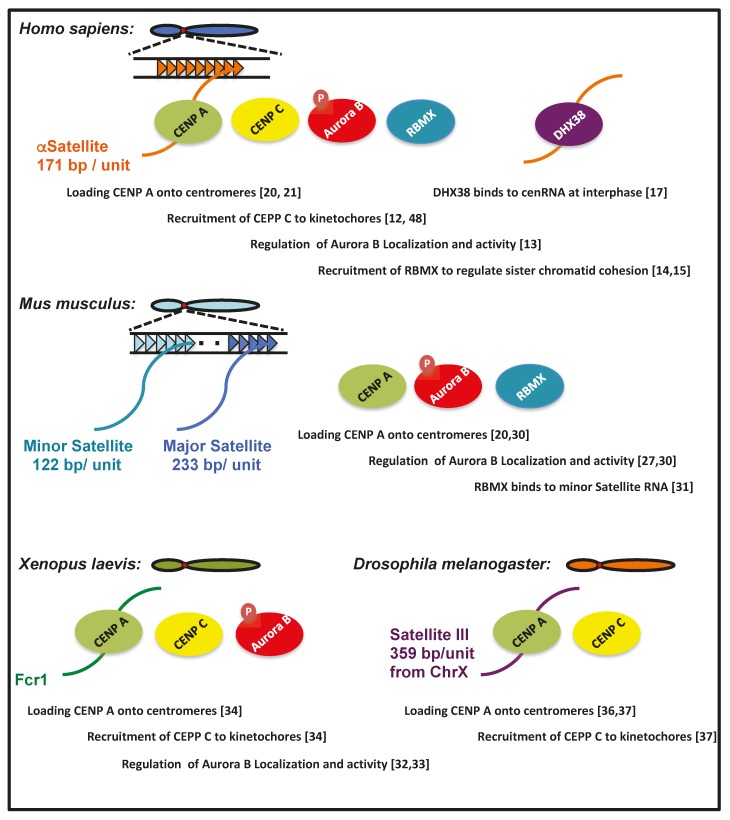
The common binding partners and functions of cenRNAs among species.

Furthermore, an RNA pulldown assay showed that CENP A also binds to mouse minor satellite RNA [30], suggesting that this RNA is included in the CENP A-containing chromatin fraction. Human cenRNA is bound by CENP A and involved in its loading to the centromere [20]. Therefore, it is likely that associations of Aurora B, RBMX, and CENP A with cenRNA are conserved between humans and mice (Figure 2).

## 3. Roles of cenRNA in *Xenopus*

RNA, which regulates the centromeric function, has also been investigated in frogs. RNase A treatment of a *Xenopus* egg extract decreased the activity of Aurora kinase B [32]. Centromeric localization of this protein was also impaired by RNase treatment [32]. IP analysis revealed that some RNA species were bound by Aurora B. Frog centromeric repeat 1 (Fcr1) RNA is one of these, which is transcribed from the flog centromeric region [33]. Antisense locked nucleic acid (LNA) against Fcr1 RNA decreased the localization of Aurora kinase B to the centromere [33]. The downregulation of Fcr1 ncRNA by the treatment with pre-mRNA splicing or transcriptional inhibitors also led to defective spindle formation in egg extracts. In inhibitor-treated extracts, the recruitment of CENP A, C, and NDC80 to mitotic chromosomes was reduced [34]. These observations suggest that the relationship between cenRNA and Aurora kinase B is conserved, even in *Xenopus* (Figure 2).

Interestingly, Fcr1 RNA could be detected not only at the site of transcription, but also in some other regions [33]. This suggests that Fcr1 RNA is free to diffuse between the centromere and is capable of binding to it in trans.

## 4. cenRNA in *Drosophila*

The *Drosophila* centromere contains AATAT and CTCTT repeats [6,35]. Pol II is detected at the centromere in the G1 and mitotic phase [36]. Nascent transcripts co-localize with CENP A at the centromere. Although the inhibition of transcription did not affect the recruitment of CENP A to chromatin, it destabilized the incorporation of CENP A into the centromere [36]. Therefore, transcription at the centromere is required for CENP A loading to nucleosomes in *Drosophila*.

Satellite III (Sat III) is a repeated sequence that comprises a 359-bp repeat unit in the *Drosophila* chromosome X centromere [37]. Transcripts of ~1.4 k nucleotides in length from this region co-localize with CENP C not only on Chr X, but also on all other chromosomes. IP analysis revealed that CENP C binds to Sat III RNA. The depletion of this ncRNA using antisense LNA caused the generation of lagging chromosomes due to defective chromosome segregation. Furthermore, the amount of CENP A and C, Spc105, and one of the KMN proteins at the centromeres was also decreased. Sat III RNA is required for the incorporation of CENP A and C into the centromere (Figure 2) [37]. Lagging chromosomes caused by the knockdown of Sat III RNA were also observed at Chr 2 and 3, suggesting that Sat III RNA derived from chromosome X could bind to other chromosomes in trans [37].

## 5. cenRNAs in Plants

cenRNAs have also been detected in plants. The maize centromere consists of satellite repeats (CentC) and centromeric retrotransposons (CRMs). These regions are transcribed to produce centromeric transcripts [38]. IP analysis revealed that the maize centromeric histone H3 (CENH3), a homologue of CENP A, is tightly bound to these centromeric RNAs [38]. Therefore, RNAs are a component of the centromere/kinetochore complex in maize. CENP C is a basic component of the kinetochore. The DNA-binding activity of CENP C is important for the function of this protein. In addition to binding to DNA, maize CENP C exhibits binding activity in single-strand RNAs. Interestingly, the association of CENP C with RNA stabilizes its binding activity [39].

The *Arabidopsis* centromere comprises 177 to 179 bp satellite repeats, called cen180. These repeats exhibit active transcription, transcripts of which are processed into small RNAs [40]. Dicer- or RNA-dependent RNA polymerase mutants are defective in the production of these siRNAs; however, no abnormalities in chromosome segregation have been detected in these mutants [40].

Further investigation is required to clarify whether the maize transposon transcripts and *Arabidopsis* siRNAs can act as cenRNA in centromeric regulation.

## 6. cenRNAs in Yeast

The centromere of budding yeast is known as a point centromere that comprises a single domain of 125 bp [6]. Its structure differs from that of centromeres in other organisms because it has no repetitive sequences [6]. In the budding yeast *Saccharomyces cerevisiae*, centromeric regions in all chromosomes are transcribed to produce ncRNAs named cenRNAs [41]. The expression levels of cenRNAs peak in the S phase. The depletion of centromere-binding factor 1 (Cbf1) and the histone H2A variant, H2A Z^Htz1^, resulted in the upregulation of cenRNAs from centromeres, leading to high rates of chromosome loss and aneuploidy. Furthermore, the knockdown of cenRNAs reduced the chromosome stability. Therefore, the optimal expression of cenRNAs is critical for chromosome regulation in budding yeast [41].

**Figure 3:**
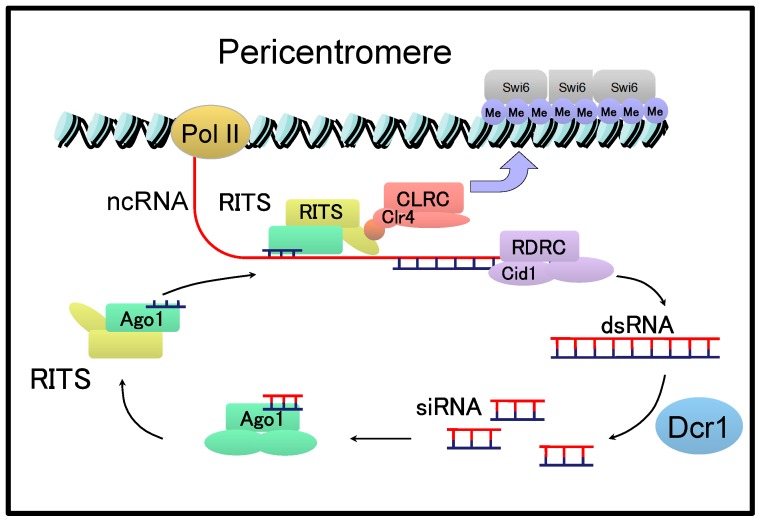
RNA interference (RNAi)-dependent heterochromatin formation at the pericentromeric region in *Schizosaccharomyces pombe*.

Different from the point centromere in *S. cerevisiae*, the fission yeast *Schizosaccharomyces pombe* has a regional centromere that spans 40–100 kb in length [42]. In the *S. pombe* centromere, a central core domain, composed of CENP A-containing nucleosomes, is flanked by pericentromeres consisting of repetitive sequences named *dg* and *dh* [42]. NcRNAs are transcribed from pericentromeric regions and are involved in the RNA interference (RNAi)-mediated formation of heterochromatin at the pericentromeres [42]. Briefly, ncRNAs transcribed from the pericentromeric regions are converted to double-stranded (ds) RNAs by the RNA-directed RNA polymerase complex (RDRC) and cleaved by Dicer (Dcr1) to produce siRNAs of approximately 22 nucleotides in length (Figure 3) [42,43].

Processed siRNAs are incorporated into the RNA-induced transcriptional silencing (RITS) complex comprising Ago1, Tas3, and Chp1. The RITS complex is then targeted to the pericentromeric region through complementarity with recently transcribed cenRNAs. The RITS complexes associated with nucleosomes recruit CLRC containing the histone-modifying enzyme Clr4p, which induces the methylation of histones and binding of Swi6, a homologue of human heterochromatin protein HP1, to form heterochromatin at the pericentromeres. Therefore, cenRNAs and their processed siRNAs play a major role in regulating the formation of heterochromatin in *S. pombe*.

Interestingly, several lines of evidence have indicated that splicing factors and introns found in the cenRNAs *dh* and *dg* facilitate the formation of heterochromatin at the centromere [18,44,45]. There is a controversial study reporting that the defective formation of centromeric heterochromatin in splicing mutants is a secondary effect that impairs the splicing of pre-mRNA-encoding factors involved in heterochromatin formation [46]. However, the following findings serve as a platform for recruiting RNAi factors to facilitate the formation of heterochromatin at the pericentromere: Deletion of the *dg* cenRNA intron significantly decreases the efficiency of heterochromatin formation in the reporter plasmid, and the physical interaction between the splicing factor, Prp16p, and a component in RDRC leads to the proposed model, in which the spliceosome or subspliceosome assembles on the cenRNA intron as a platform [18,45].

In addition to the pericentromeric region, central core domains, possessing CENP A at the centromere, are transcribed by Pol II into ncRNAs. Since these ncRNAs are rapidly degraded after transcription by the exosome system, it has been suggested that transcription itself, not transcripts, may play an important role in CENP A loading in the central core domains [47]. However, it currently remains unclear whether the transcripts from central core domains also play roles as unstable long or small ncRNAs in centromeric functions.

## 7. Conclusions

Numerous protein factors are thought to be involved in centromere regulation [1,6,7]; however, the mechanisms underlying the interactions of these factors have not yet been elucidated. In this review, we have described previously reported cenRNA roles in centromere function among different species, in addition to the protein factors. Understanding how cenRNA regulates centromere function can help clarify how this region functions in a complicated manner to regulate the dynamics of chromosomes.

**Figure 4:**
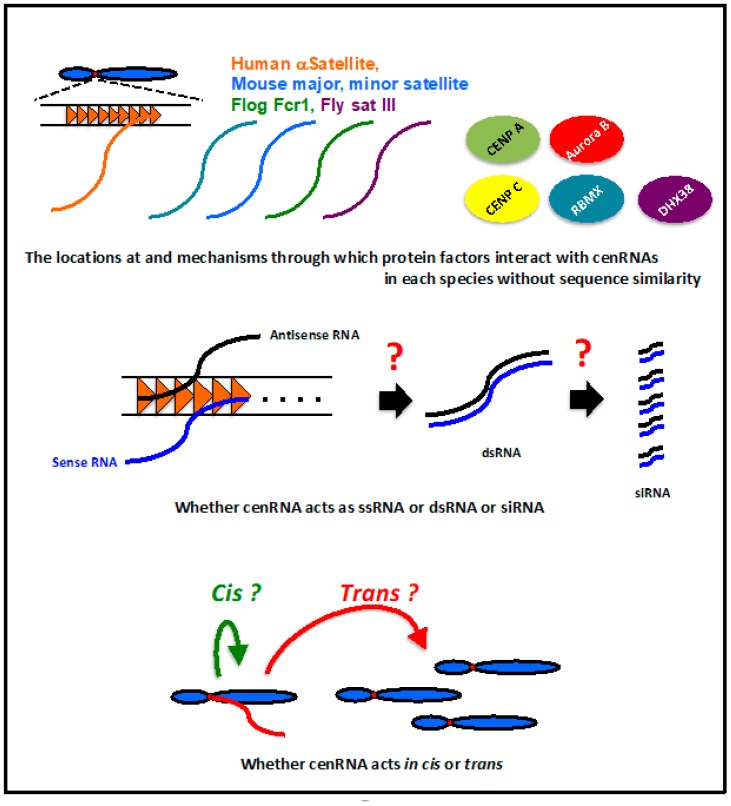
Unsolved questions about cenRNAs.

In humans, mice, flies, and frogs, cenRNAs are involved in CENP A loading into centromeres, the association of CENP C to kinetochores, and regulation of the localization and activity of Aurora kinase B at the centromere. Even though these regulatory activities are very important for centromere function, the mechanism underlying their regulation by cenRNA is not clear. Importantly, whether these factors interact directly with cenRNAs is still unknown. The RNA binding ability has been reported for CENP C [39], but not for CENP A and Aurora B. Because cenRNA has no sequence similarity among species, the binding site of these factors may be species-specific. These factors may interact with cenRNA, together with other protein factors. The locations at and mechanisms through which these factors interact with cenRNAs in each species should be elucidated (Figure 4).

In addition to the abovementioned proteins, RBMX was identified as a common cenRNA interacting factor in humans and mice [14,31]. cenRNA may form RNP with some protein factors. Although our research group and other groups have tried to identify the cenRNA binding factors in humans and mice, the whole components of cenRNP remain to be determined. The identification of the whole cenRNP composition and a comparison of it among species might be able to help researchers understand several of the aforementioned problems.

The mechanism underlying the regulation of centromeric factors by cenRNA is also very important. Although cenRNA controls the kinase activity and localization of Aurora B [13,30,32,33], the molecular basis of it has not yet been elucidated in any organism. The regulation of CENP A loading and CENP C association with the centromere by cenRNA is also unclear. For example, if we can determine the crystal structure of cenRNA-centromere factors, it may lead to a solution.

cenRNA function in the cell cycle should also be studied. The detailed expression or accumulation pattern of cenRNAs among various species is also unavailable. Because the timing of CENP A loading into the centromere varies between species [4], the expression and functioning duration of cenRNA should be well-controlled in each species.

Human cenRNA even accumulates at the interphase. The composition of cenRNP is different between the interphase and mitotic phase [14]. This suggests that RNP remodeling occurs during cell cycle progression. The specific cenRNA composition may play a substantial role in this phase. In HeLa cells, a relationship between cenRNA and the ICEN complex has been suggested [17]. Although the function of this complex is still unknown, it is interesting that the complex containing CENP A and many other centromeric proteins is formed in the interphase. In addition, αSatellite RNA accumulates in the interphase nucleolus with CENP C1 and INCENP [48]. The roles of cenRNAs in the interphase warrant further studies.

Human cenRNA was detected from both strands [13]; however, it is unknown whether these transcripts form ds RNA. Whether cenRNA acts as a long RNA or processed short (small) RNA remains to be determined (Figure 4). The regulation of heterochromatin formation using siRNA derived from the pericentromeric region in *S. pombe* is unique [42]. However, there is limited evidence to show that RNAi machinery is also involved in the centromere regulation of higher eukaryotes. The loss of Dicer activity results in the accumulation of long transcripts from human satellite repeats in a chicken–human hybrid DT40 cell line [24]. A small RNA species derived from a major satellite region was detected in mouse cells [29]. However, further clear evidence is needed to determine if cenRNA functions as an siRNA. In case cenRNA actually functions as an siRNA, elucidation of the mechanism and pathway for siRNA generation is very important. 

Whether cenRNA remains at the transcription site or is released from there is not clear. If cenRNA is released and localized elsewhere, the mechanism underlying this should be determined.

It is difficult to determine whether cenRNA is produced from all the centromeric regions equally on every chromosome. Therefore, cenRNA binding to the chromosome in cis or trans is an important issue (Figure 4). In *Drosophila*, sat III RNA transcribed from chromosome X can bind to other chromosomes in trans [37]. Frog cenRNA, Fcr1 RNA, was detected at other centromeres, suggesting that this RNA is capable of functioning in trans [33]. There is no clear evidence that shows human cenRNAs acting in trans. The expression from plasmid-coding satellite I sequences did not lead to any abnormal phenotype in HeLa cells [13]. This result may suggest the possibility of cenRNA not functioning in trans in human chromosomes. In mice, the expression of major satellites from plasmids leads to mislocalization of Aurora B to the centromere and a lack of sister chromatid cohesion [27]. However, more investigations are required to clarify whether the mouse cenRNA acts in trans.
